# Acute Abdominal Compartment Syndrome following Extraperitoneal Bladder Perforation

**DOI:** 10.1155/2017/3073160

**Published:** 2017-05-30

**Authors:** Ana Licina

**Affiliations:** Austin Health, 145 Studley Road, Heidelberg, VIC 3084, Australia

## Abstract

Extraperitoneal bladder perforation is a known complication of a commonly performed rigid cystoscopy. If unrecognized, this complication can lead to continuous intra-abdominal fluid leakage with consequent organ function impairment and symptoms. This is the first case report in literature of a transurethral bladder perforation causing an acute abdominal compartment syndrome, which was subsequently managed conservatively with supportive management only.* Case Presentation.* We describe a clinical course of a 73-year-old Caucasian female whose initial acute presentation involved urinary symptoms. Surgery and general anaesthesia during rigid cystoscopy were complicated by an initially unrecognized extraperitoneal bladder perforation, resulting in fluid extravasation. This extravasation resulted in transurethral bladder resection syndrome with acute intra-abdominal free fluid accumulation. This complication caused acute abdominal compartment syndrome resulting in respiratory end-organ compromise and immediate postextubation respiratory failure. Patient required an emergency reintubation. During the management, diagnosis was considered through the use of the point of care abdominal ultrasound. Postoperatively, patient was managed conservatively in intensive care. Postoperative course included an approximate nine liters of urinary diuresis and supportive ventilation for four days.* Conclusion.* There is equipoise in the clinical management of abdominal compartment syndrome with regard to supportive medical management alone or invasive surgical treatment.

## 1. Introduction

Asymptomatic bladder perforation during a rigid cystoscopy and transurethral bladder tumour treatment may occur in up to 58% of patients [1].

If severe, intraoperative bladder perforation can result in intra-abdominal fluid accumulation causing intra-abdominal hypertension (IAH). IAH is defined as a sustained intra-abdominal pressure greater than 12 mmHg [2].

If IAH is severe, it can result in abdominal compartment syndrome.

We describe the clinical course of a patient who experienced an unrecognized extraperitoneal bladder perforation, leading to massive intra-abdominal fluid accumulation, IAH, and subsequently ACS.

In this case, acute IAH caused immediate respiratory failure on extubation due to a mass effect of fluid and subsequent lung compression.

Further notable features of this case are the physiological changes related to the perforation of the bladder with a fluid overload pattern and electrolyte derangement distinct from the transurethral prostate syndrome [3]. We note the differences between the two in the Discussion.

## 2. Case Description

This seventy-three-year-old Caucasian female with a prior history of endometrial cancer and suspected recurrence demonstrated on CT abdomen and pelvis was booked for a rigid cystoscopy and urgent bilateral ureteric stent insertion.

This investigation demonstrated a potential localized recurrence of the tumour with bilateral compression of the ureters resulting in bilateral hydronephrosis.

Her past medical history included a hysterectomy, radiotherapy, and chemotherapy for endometrial carcinoma 2 years ago. Other comorbidities included noninsulin dependent diabetes, hypertension, and body mass index of 39. Renal function was acutely impaired with Cr = 200 *μ*mol/l and estimated GFR was 30 mL/min/1.73 m2.

Uneventful modified RSI was performed with a size 7 standard endotracheal tube for airway management and maintenance. During the course of surgery, anaesthesia was initially uneventful. Approximately thirty minutes from the start of the procedure, there was an unexplained rise in airway pressures from an airway peak pressure of 35 to 40.

This coincided with the neuromuscular paralysis wearing off and the patient was given a further dose of atracurium. High airway pressures moderated with paralysis and an alternative ventilation strategy.

Bilateral ureteric stents were inserted with real-time radiological guidance and correct positioning was confirmed. Throughout the procedure, ongoing bladder washout was continuing with the irrigation fluid containing glycine 1.5% with a total volume of ten liters used. The washout solution was hung at standard height of two meters and rate of infusion controlled by the operating surgeon. The procedure was deemed technically difficult and took ninety minutes of surgical time to complete.

Patient was extubated once all extubation-readiness criteria were met. Immediately after the endotracheal tube (ETT) was removed, patient became agitated, diaphoretic, and progressively centrally cyanosed. Haemodynamic instability was noted with severe hypertension with systolic blood pressure reaching 200 mmHg.

Decision was made to reintubate the patient.

Once the ETT was resecured uneventfully, the usual algorithm for respiratory distress was followed and definitive diagnosis for severe diaphoresis was not made [4].

Further large bore intravenous access was obtained and 20 g radial arterial line was inserted. The patient was examined in her entirety and the expanding abdominal girth was noted by the theatre team. Ultrasound fast scan was performed and a significant amount of free fluid was noted surrounding the spleen and the left kidney.

**Figure 1:**
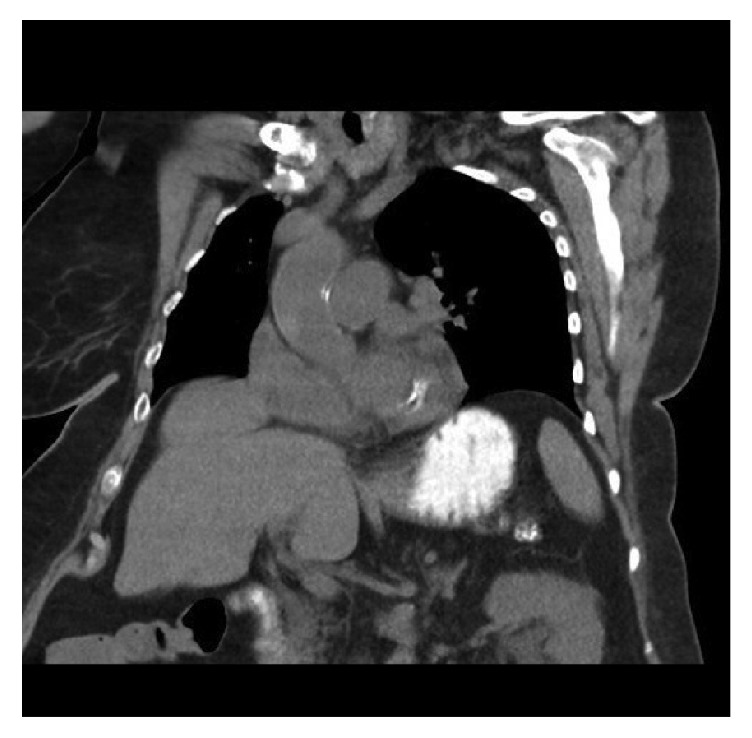
Sagittal CT scan demonstrating no free fluid in the abdomen.

Comparison was made with the CT scan obtained 24 hours prior to the procedure, where no free fluid in the abdomen was seen (Figure 1).

The washout solution at this stage was changed to normal saline and rate was decreased as per surgical team instructions.

First blood gas on 100% oxygen after reintubation and arterial line insertion demonstrated hyponatraemia: pO2 = 184 mmHg, pCO2 = 54 mmHgm, Na = 126 mmol/L, K = 3.8 mmol/L, and HCO3 = 21 mmol/L.

**Figure 2:**
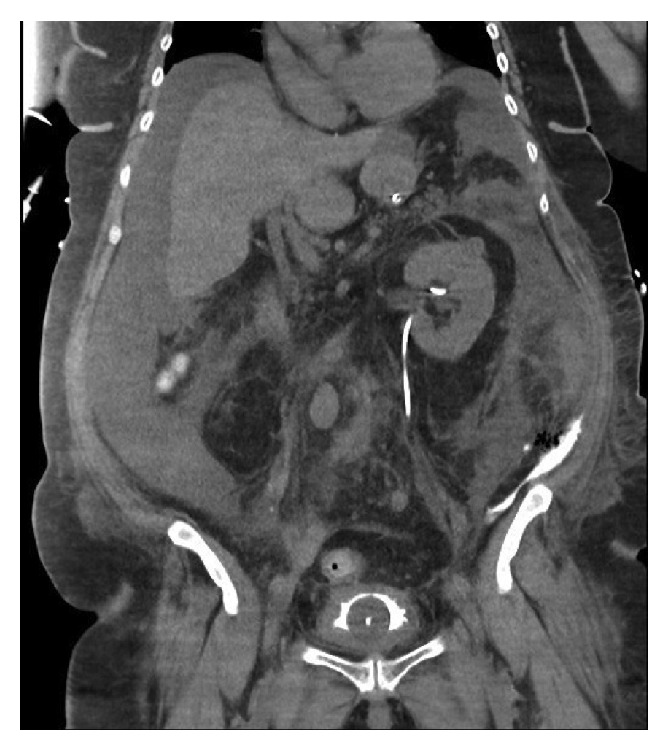
Sagittal CT scan illustrating fluid accumulation around the liver and in the R paracolic gutter.

CT scan chest/abdomen and cystogram confirmed a bladder perforation and intra-abdominal fluid extravasation as illustrated in Figure 2. Continuous bladder irrigation was ceased and patient was taken to intensive care intubated.

Supportive management was continued in intensive care and she remained intubated for further 4 days. Supportive medical management included respiratory system support through artificial ventilation, sedation facilitating ongoing intubation and improving the abdominal wall compliance. After the initial episode of hypertension at reintubation, there was no further haemodynamic instability.

Diuresis was achieved through use of daily loop diuretics. Electrolytes were carefully monitored. As there were no clinical signs of hyponatraemia, hypertonic saline was not used. There was no abdominal percutaneous tap performed in order to drain the fluid and there was no exploratory laparotomy performed either. As expected, this patient developed pulmonary oedema with clinical evidence on CXR and impaired gas exchange. Eight and a half liters of fluid was diuresed during the intensive care stay. Patient was extubated uneventfully and discharged to the ward.

After an initial persistent hyponatraemia, sodium returned to normal values in 7 days from the precipitating event.

## 3. Discussion

There are various definitions of abdominal compartment syndrome—a research definition which states that ACS is defined as a sustained intra-abdominal pressure > 20 mmHg (with or without abdominal perfusion pressure < 60 mmHg) that is associated with new organ dysfunction [2]. In clinical practice, although desirable, it is not always possible to measure the intra-abdominal pressure [5].

For clinical purposes, intra-abdominal compartment syndrome is better defined as intra-abdominal hypertension (IAH) induced new organ dysfunction without a strict intra-abdominal pressure threshold, since no intra-abdominal pressure can reliably diagnose all ACS [2].

Recognition of the acute IAH prior to the development of ACS is preferable as prompt treatment of the underlying cause can decrease the end-organ complications [6]. Development of abdominal compartment syndrome is the end of a pathophysiological spectrum, which is on a continuum of steady increases in intra-abdominal pressure [7]. At the severe end, it encompasses multiple biomediator generation which can result in multiorgan dysfunction syndrome.

In acute ACS, clinical symptoms can include any one of the following: cardiovascular, pulmonary, renal, hepatic, gastrointestinal, and central nervous system complications. Cardiovascular instability occurs due to the external pressure exerted on the vena cava significantly decreasing the venous return and cardiac output [8]. Respiratory system is affected due to a functional restriction of the diaphragmatic excursion. This results in significantly decreased respiratory compliance, hypoxemia, and CO2 retention.

Abdominal distension in this case affected both respiratory and cardiovascular systems. Although abdominal distension was not fully clinically recognized until the patient had failed extubation, diagnosis of ruptured bladder occurred after the respiratory failure and clinical ACS development.

The anaesthetic team noted raised airway pressure during anaesthesia. The standard approach of simultaneous diagnosis and management was taken but had a negative clinical yield. Increasing airway pressures are one of the known clinical signs of ACS [9]. This occurs due to extravasated intra-abdominal fluid having an effect on the respiratory function due to local compressive effect affecting functional residual capacity and causing significant bilateral lower lobe lung collapse.

There had been a failure to diagnose the abdominal expansion at this point. The patient had been covered with a warming blanket in a lithotomy position, and the anaesthetist did not conduct an abdominal examination.

A crude examination of the abdomen would have been helpful in this case.

It has been suggested that measurement of the abdominal girth and examination of the abdomen may be helpful when querying a bladder rupture. A more helpful approach in these cases may be to routinely examine the abdomen in patients having a rigid cystoscopy, particularly considering the high rate of asymptomatic bladder integrity compromise [10]. In a case illustrating acute ACS after a transurethral resection of the prostate, authors note the change of practice in urological procedures: they make a point of routinely assessing the abdomen visually with operating room lights on before extubation [10]. They also note newly implemented measurement of the input and output during the urological cases. Difficulty with ventilation can alert the anaesthetist to consider fluid extravasation in any endoscopic bladder procedure or arthroscopic hip procedure [10, 11].

In this case, bladder perforation was diagnosed due to the above respiratory presentation, failure to identify primary respiratory pathology, and, on a secondary survey, a rapidly expanding abdomen. Clinical suspicion was confirmed through the use of the FAST ultrasound scan, which demonstrated fluid in the abdomen and the subsequent imaging with the use of a CT scan. Clinical utility of ultrasound in this case was high. Anaesthesia as a specialty has recognized the importance of competent use of ultrasound in various diagnostic and management applications.

We have touched on some of the pathophysiological processes including local compressive effects on the cardiovascular and respiratory system as well as the biomediator generation resulting in acute intestinal distress and multiorgan failure [7]. Some of the main risk factors for development of ACS include damage control surgery in trauma patients. Others include intra-abdominal procedures, abdominal trauma, ruptured abdominal aneurysm, haemoperitoneum, liver transplant, and fluid extravasation after endoscopic procedures.

Management of ACS is broadly divided into supportive medical management strategies and more invasive surgical management [12]. Supportive medical management includes essential cardiovascular support as well as bowel lumen decompression, diuresis and improving of the abdominal wall compliance with sedation, and muscular paralysis.

There is still limited evidence as to whether treatment of IAH and even ACS in critically ill patients improves patient outcomes [7]. Well-designed studies comparing medical management of ACS with surgical decompression need to be performed as there is equipoise amongst clinicians as to the need for decompressive laparotomy in some conditions.

In this case, decision was made to not perform a postoperative laparotomy or a paracentesis. Either can be considered as definitive management of the surgical complication and free water release. This management decision was based on the successful results of supportive medical therapy including improving the abdominal wall compliance and diuresis. A supportive percutaneous tap was not considered by the management team at the time. This may have been beneficial due to decreasing the amount of fluid requiring diuresis and decreasing the physiological disturbance. In prior case reports of transurethral bladder perforation, all patients with extraperitoneal rupture were treated with an urgent laparotomy [3].

Intra-abdominal pressure in our patient was never measured; however with elevated airway pressures, respiratory failure due to localized compressive effect, and an eight-liter fluid diuresis, it is likely that this threshold was achieved. There have been alternative algorithms suggested [11]. In a recent case report, intra-abdominal extravasation occurred during hip joint laparoscopy. The authors suggested an algorithm use for patients suffering from intra-abdominal compartment syndrome during a hip joint arthroscopy. Following this algorithm, our patient could have been treated with abdominal paracentesis. Considering the similar pathophysiology of fluid extravasation, this may be an appropriate algorithm to follow in extraperitoneal bladder perforation and significant fluid accumulation.

There are previous short case series reports in the literature of intra-abdominal fluid accumulation following a bladder perforation [3]. All of the cases were recognized prior to completion of the surgery. All of the patients experiencing extraperitoneal perforation with severe symptoms were managed proactively with a surgical laparotomy. During a further literature review of cases, there have been reports of ACS following bladder neck rupture while performing a TURP [10]. In that situation, patient was managed with a laparotomy and had three liters of fluid extravasated in the abdomen in contrast to the more significant extravasation of eight to nine liters.

This patient suffered from a transurethral bladder syndrome, which results from the absorption of fluid down the osmotic gradient across the permeable peritoneal membrane. In contrast, in TURP syndrome, fluid absorption enters the intravascular circulation directly through compromised vessel integrity in a more immediate fashion.

TURP syndrome involves more rapid direct extravasation of fluid into the intravascular space. The full metabolic effects of intraperitoneal fluid absorption due to bladder perforation are therefore delayed. There has been a delineation between “TURBT” syndrome with the absorption of large volume of free water across the peritoneal membrane and “TURP” syndrome with its immediate consequences of intravascular overload.

Table 1 illustrates the differences in fluid pathophysiology with transurethral bladder perforation versus transurethral prostate perforation.

Sterile nonpyrogenic 1.5% glycine is preferred by urologists as it is nontoxic, nonhaemolytic with a refractive index close to that of water. When metabolised, glycine is transaminated to serine and deaminated to ammonia which is converted to urea [9]. Systemic absorption of large volumes of glycine results in the metabolic consequences of free water absorption and metabolism of ammonia to urea. This results in hyponatraemia due to a dilutional effect. There is also movement of sodium into the peritoneal fluid across the osmotic gradient, which results in greater hyponatraemia with potential for confusion and seizures [13, 14].

Free water overload distributes equally across the intravascular and extravascular components. Clinical expectations are those of TURP-like syndrome [3].

Lowest sodium was observed in the immediate postoperative course with the value recorded of 125 meq/ml. This reflected a drop of 10 units, where the immediate pre-operative sodium was 135 meq/ml. The value took 7 days to normalize, with the return to 137 meq/ml.

## 4. Conclusion

We have presented a case of primary acute abdominal compartment syndrome occurring intraoperatively, which was managed entirely through medical supportive treatment. There are suggestions that equipoise exists as to the need for surgical versus medical management in abdominal compartment syndrome, with a need for further valid studies.

As extraperitoneal fluid extravasation may occur during rigid cystoscopy, anaesthetists should consider monitoring clinically the abdominal girth routinely during these cases as well as use of ultrasound to assist their diagnosis.

It is important to differentiate clinically transurethral bladder perforation to transurethral prostate syndrome. Pathophysiology of the fluid entry and overload is different leading to alternative optimum medical management of either syndrome.

## Figures and Tables

**Table 1:** Differences in resulting pathophysiology between transurethral bladder perforation and transurethral prostate perforation.

	Transurethral bladder perforation	Transurethral prostate perforation
Mode of fluid absorption	Absorbed across the peritoneal membrane	Direct intravascular entry
IAH with potential ACS	Yes	No
Fluid compartment affected	Extra- and intracellular, intravascular	Intravascular
Respiratory	Atelectasis due to abdominal girth expansion, pulmonary oedema due to TBW overload	Pulmonary oedema due to intravascular fluid overload
Cardiovascular	Relative hypovolaemia, hypotension	Hypertension and bradycardia followed by hypotension
Neurological	Decreased GCS	Decreased GCS
Gastrointestinal	Decreased perfusion due to ACS and hypotension	Unlikely to be affected

## Abbreviations

IAH: Intra-abdominal hypertension
ACS: Abdominal compartment syndrome
TURB: Transurethral bladder
TURP: Transurethral prostate
ETT: Endotracheal tube
TBW: Total body water.
